# Editorial

**Published:** 1858-09

**Authors:** 


### Editorial.

### GALVANISM IN EXTRACTING TEETH.

This subject is exciting considerable attention just now, and very properly too. The method of its application has been so often described, that it seems scarcely necessary to refer to it here, a few words, however, may not be out of place.

Any ordinary battery will answer the purpose. The one most convenient is that manufactured by Neff, of Philadelphia, but any manufacturer can make them just as well. I have used the ordinary physician's battery, that has been in use a dozen years or more, with just as good results as any other.

The attachments should be made in the most convenient manner. The instructions are to attach the negative pole of the battery to the forceps, and the positive pole to the hand of the patient, so that the current will pass through the arm, and tooth to the forcep. It is, notwithstanding this direction, a matter of no consequence, which pole is attached to the forceps, the results according to experiments, are just the same, it could not be otherwise. A foot board, at the command of the operator for breaking the connection is a good arrangement, but it requires some practice to use it with facility. The connection should be made the moment the tooth is to be extracted, after the forcep is placed upon the tooth.

The effects.—In almost every case, where it has been employed, the persons refused to have teeth extracted a second time without it, yet in no case has it prevented all pain, during the operation, but all concur in saying it mitigates it very much, but upon what principle I can not determine. The probability is, that the nervous system is sustained by the galvanic current, so that the shock is not so great, under the influence of the operation: the mind is also diverted. The strength of the current required, will be different in different persons. That will be very easily determined by the patient holding the poles of the battery, in the hands, and thus testing the amount they can bear; too great an amount will produce pain independent of the extraction of the tooth. No greater amount should be given, than can be borne by the patient comfortably. There are no subsequent ill effects, at least I have observed none, and can not conceive any. The forcep should be isolated from the hand of the operator, by a silk or kid glove; and should be guarded, so as not to touch the lips or tongue of the patient, as it produces a very disagreeable sensation; in a small mouth it is best to lay a small napkin, on the lips to protect them. The forcep should also come in contact with the gum as little as possible.

### REGISTER—BACK NUMBERS.

We are anxious to get a few copies of the December No., 1856, the second number of Vol. X. Any persons having this number, and are not anxious to retain it, will please forward to us, we will give one dollar each, for fifteen to twenty copies, or will give two of the other numbers for one of this. Please send along.

### OHIO DENTAL COLLEGE.

The fourteenth regular annual session of the Ohio College of Dental Surgery, will commence on the first Monday of November next, and continue until the twentieth of February following. Preliminary lectures will be delivered daily on and after Monday, Oct. 18th. It is very important that students be in at that time. The Labaratory and Infirmary will be open at that time.

We hope to see all the members of the class in promptly.

### ALLPORT'S DENTAL LEDGER.

We are now using a copy of Dr. Allport's Dental Ledger, and find it quite an acquisition. By using it your books are always posted; and at the same time you have a record of all your operations. Like all highly useful things, it is very simple in its design, and convenient in its application. It is not, we believe, copy righted; and Dr. A. informed us that any publisher was at liberty to use the design.

### CORRECTION.

At our request, the article in the last number, entitled "Local Anæsthesia," was translated for us from *L'Art Dentaire*, by our friend COATES-KINNEY; but our ascription of the translation to him (not at his request, however) made him responsible for a few errors that might disparage his scholarship with the reader, if the errors were his—but they were not; for in his manuscript we find, what we should have found before if we had carefully collated the proof, as follows: "poudre," for "powdre;" "edematous pad," for "edemacious pod;" "gramme," for "gramine;" "syphillitic," for "sympathetic."

---

[OUR friend whose initials lie at the bottom of the following *pome*, has pæanized the triumphs of science in high style. Whatever other merits it may claim, our profession can not set up a title to *poetry;* yet "I. B. B." has here demonstrated that *verses* can be written on some subjects as well as others. Take, for instance, the line,

"Or, if we're afraid lest we may *wake dead*,"—

in which the italics are ours—and observe the correctness of the measure and the allowableness of the rhyme, saying nothing of the extremely disagreeable predicament in which the supposed "we" wake and find themselves. Shades of Sennacherib's host, what a fix! However, we need not say that, in spite of the little faults, our friend's machine has turned out a first-rate article; for, zounds, do you think we would publish it else?]

### HO, HO! FOR THESE DAYS.

Ho! ho! for these days, and these modern ways,
  When science so rapidly rides,
That the fastest of men, with their keen ready ken,
  Can hardly keep pace with her strides—
When steam, frost, and light, have become her delight,
  And work at the bidding of man;
When the lightnings are chained, yet have never complained,
  But patiently do what they can;
When o'er the sea's bed the nuptials are said,
  That marry the nations together,
And the electrical flash is an order for cash,
  Or a note on the state of the weather;
When if, as you pass, you look in the GLASS,
  The light has your features at once—
Your picture is there, at which all may stare,
  And call you a knave or a dunce;
When the lucifer match, with a most gentle scratch,
  Takes place of the flint and the steel,
And the strong Iron Horse, with terrible force,
  Treads Time with his scythe 'neath his heel;
When children are fed, by bumps on their head,
  To bid their vile passions be still,
And steel and fine gold in the market are sold,
  Instead of the "gray goose quill."

Ha! ha! for these days, when science displays,
  Her power o'er the tortures we feel;
No longer, per force, we bear them of course,
  Because we're afraid of the steel;—

But instead of the pangs, from the wrenching of fangs,
Or the cutting of nerves all diseased,
We now go to sleep—and wake not to weep,
But to smile that the horror has ceased.
Or, if we're afraid lest we may *wake dead*,
As indeed too oft is the case,
Then the "*Local*" we try, with the frost in his eye,
And soon the offender displace;
For he in a trice cools it all off so nice,
We laugh at the surgeon, forsooth;
And scarce will believe, till we feel and perceive,
We're minus an old aching tooth.
Are we fond of new things, then we just hold the strings
To the engine of lightning at play,
And feel the quick claws hook into our jaws,
And forget while the tooth breaks away.
Ha! ha! for the age, when history's page
The wonders of science contains:
May the wonders grow as the ages go,
Till the Light o'er the Darkness reigns.

I. B. B.

---

### THE WESTERN DENTAL SOCIETY.

The third annual meeting of this society was held in Quincy, Ill., on Tuesday, the 20th of July. From the report which we have heard a good time was enjoyed. We have not been able as yet to procure a report of the proceedings and discussions for publication. We attach blame to no one but ourselves, for this, it was impossible for us to get them and make a report for the Register.

Dr. Leslie of St. Louis, made a full report, which will be published in the "Dental Review;" (by the way it is due before this,) there was a fine list of subjects, and ample justice was done to them.

It makes one happy to see the whole-souled way in which our Western brethren do up *business*. The unanimity is truly refreshing. There are some symptoms that we have caught a slight *snuff* of it here in Cincinnati, but it is not noised abroad much yet, hope the seed won't *rot*.

All who wish to see the discussions and proceedings, just subscribe for the "Dental Review," and pay a *dollar*.

---

### PRIZE ESSAY.

The second edition of the Prize Essay has been issued, in three styles, bound in paper, flexible cloth, and stiff binding and full gilt, at six, twelve, and twenty-five dollars per hundred respectively.

It is beautifully gotten up.

Orders can be supplied by addressing Drs. Richardson and Taft.

It is a work with which much may be done, by a free distribution.

### AMERICAN DENTAL CONVENTION.

This body met in this city on the 3d of August, and continued in session till the 6th. It is unnecessary to say to those who were here, that it was a good time.

In some respects there was much improvement.

The discussions were certainly of a higher order than heretofore. This no doubt resulted very much from the publication of the subjects for discussion previous to the meeting.

The members had evidently studied the subjects, and came prepared to give their views definitely and concisely upon the various topics, and they did it, as the report, imperfect as it is, we think, shows.

There was no element of discord or dissension entered into any of the proceedings, but all went harmoniously on, throughout. A great many new ideas were advanced, some of them very valuable, and a few *otherwise.* There was no startling new developments, but many new particulars, and shades of difference in practice, and special manipulations, that will be very valuable to those who heard them, and improve thereby. We think that on this account there are many others who should have been there. Now, gentlemen of the profession, you who stayed away because you thought you would not be profited; is there nothing more that you can learn, in the practice of your profession? and if there is not in the practice, is there not in the science of it? Nineteen-twentieths of those *exclusives*, have neither the practice nor the science of the profession in any good degree.

Exclusive, hide-bound, hanging like a dead member to a living body, to be dragged along in disgust.

"Oh, we get it all in the reports in the journals any-how." *No you don't*, and what little you do get, you take it without giving any thing in return.

These remarks are applicable to those only who do nothing for the promotion of the profession, and we do wish somebody would give them a "*good swipe.*"

---

### A BLUNDER.

Our printers made sad work with Dr. Muse's article on "Cheoplasty," in the June number of the Register, page 375; page 376 is without beginning or end, so far as it appears there. In order to read it all right, immediately after the word Cheoplasty, page 375 end of the fourth line from the bottom, read page 376 and close with the last three lines of page 375, and it will come out straight. It is not quite so bad as the Dutchman's horse.

Dr. Muse, you can't be more vexed with that kind of a "*fix up*" of your article than we, so keep quiet and write another and we will do better.

### THE NEW YORK DENTAL JOURNAL.

Edited by F. H. Norton and G. W. Perrine.

The first number was issued on the first of July last. It is a neat Journal well gotten up, and its pages filled with interesting matter. It certainly gives evidence of editorial ability.

We welcome the new comer with pleasure, and hope its career will be one of usefulness to the profession, and emolument to its proprietor. May it never *grow* less, but *swell.*

---

### THE REGISTER.

The proprietorship, and publication of the Dental Register has passed wholly into the hands of Dr. J. Taft, to whom all communications and business letters for the Register must be addressed in the future.

Dr. Watt will remain as one of the Editors for the present at least.

---

### A SUGGESTION.

If any of our subscribers wish to pay for the Register, they can do so without any fear of making *us mad.*

---

Thanks to our Amanuensis, for assistance with this number of the Register.

---

### BILLS.

We send out bills with this number to our delinquent subscribers, with the hope that they will forward the amount due immediately. We have received on subscription for the eleventh volume, only a fraction over half enough to pay the printer; the balance, several hundred dollars, we have advanced from our own pockets. Now is this as it should be, we give you our time, and you receive a journal, that we hope is of some professional interest to you—if it is not, send it back—and yet we do not receive enough from this energetic, go-ahead, wealthy profession, to pay for the printing.

We have, since our last issue, cut off about two hundred names of delinquents from our list, and there are a host left; well, we intend to continue to cross till we get a paying list. We are constantly improving the Register, and getting less for it.

We hope all will remember that the terms are advance payment, and it is easier to pay at the beginning, than at the end of the year. Please try it.

